# Supplementary material for: Validity of Ultra-Short-Term HRV Analysis Using PPG—A Preliminary Study
Source: Sensors (Basel). 2022 Oct 20;22(20):7995. doi: 10.3390/s22207995 (PMC9611389; doi:10.3390/s22207995)
Supplement: Supplementary file 1 [file sensors-22-07995-s001.zip › Figure S1. Original Bland-Altman plots for a selection of HRV features extracted from PPG.pdf]

Figure S1. Original Bland-Altman plots for a selection of HRV features extracted from PPG recordings of 5 min 30 s and 1 min 30 s durations and 25 Hz and 200 Hz sampling rates (HRV^{F}_{PX}), compared with the HRV features extracted from ECG recordings of 5 min 30 s duration (HRV_{E5}). All HRV features were transformed with the log transformation before plotting the Bland-Altman plots. The selection of the features to be visualized was based on the results presented in Table 2 of the paper and on the correlation coefficients between the valid features. Therefore, only features that were valid in ultra-short recordings and that were not correlated (correlation coefficient < 0.9) with other features were shown hereafter.

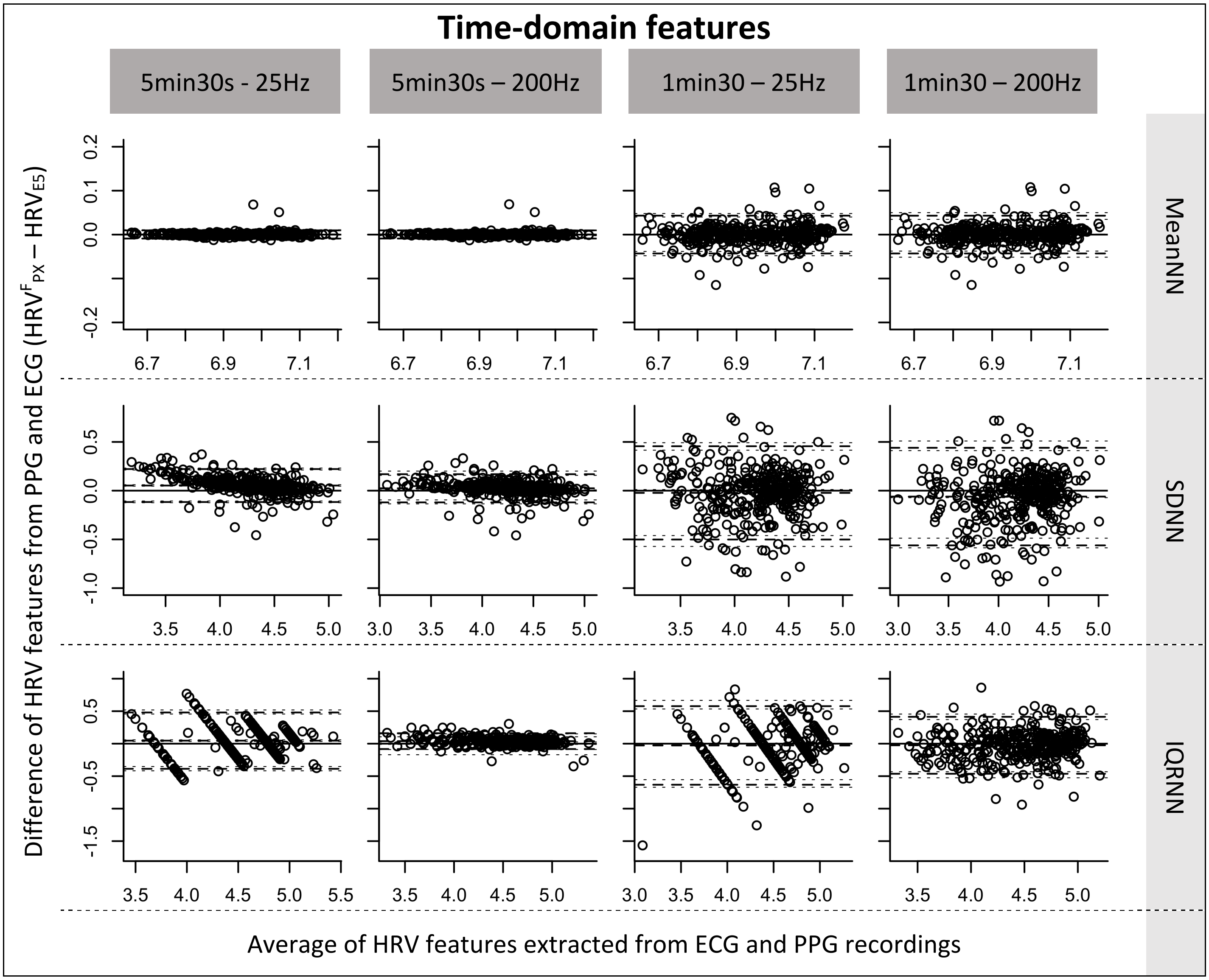

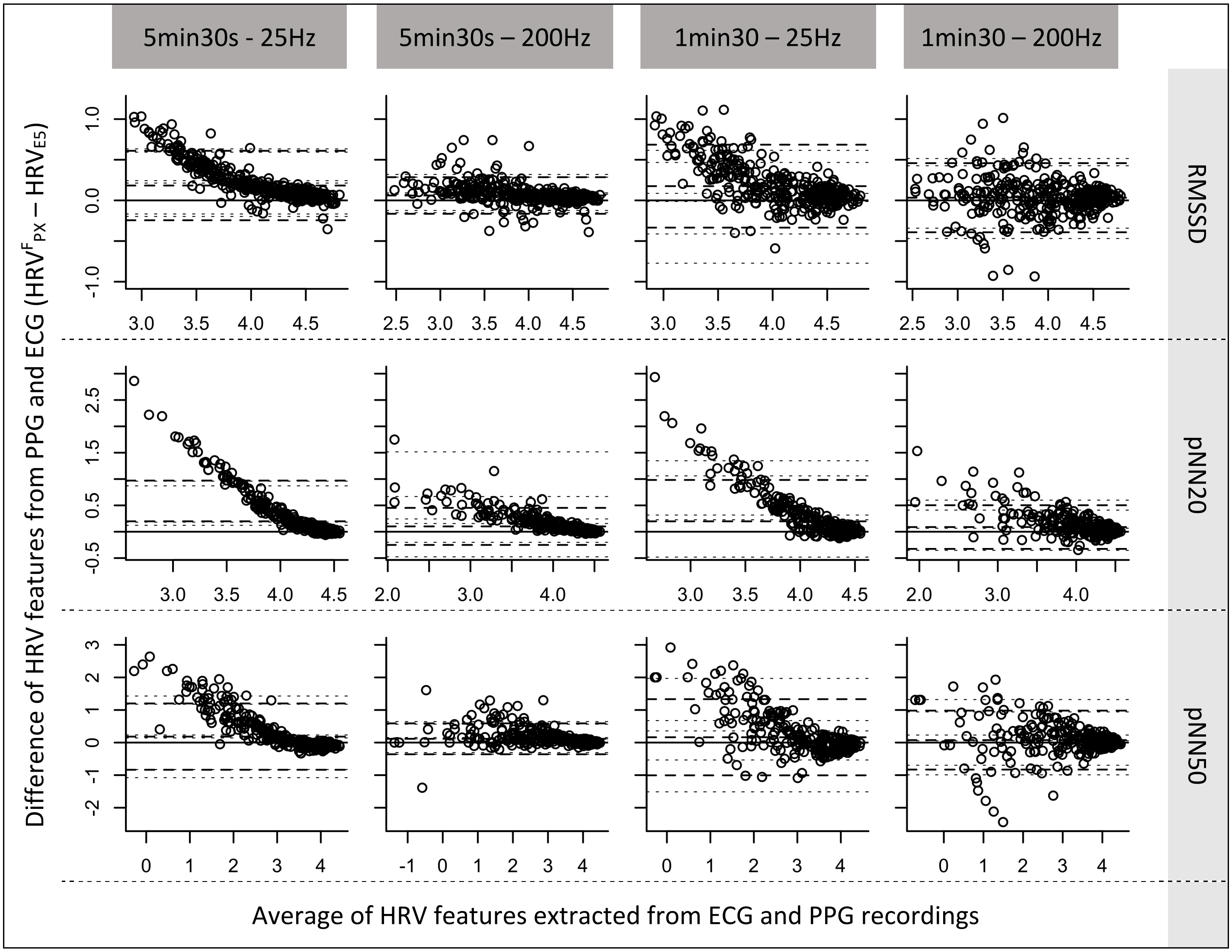

5min30s - 25Hz
5min30s – 200Hz
1min30 – 25Hz
1min30 – 200Hz
RMSSD
pNN20
pNN50
Difference of HRV features from PPG and ECG (HRV^F_PX – HRV_E5)
Average of HRV features extracted from ECG and PPG recordings

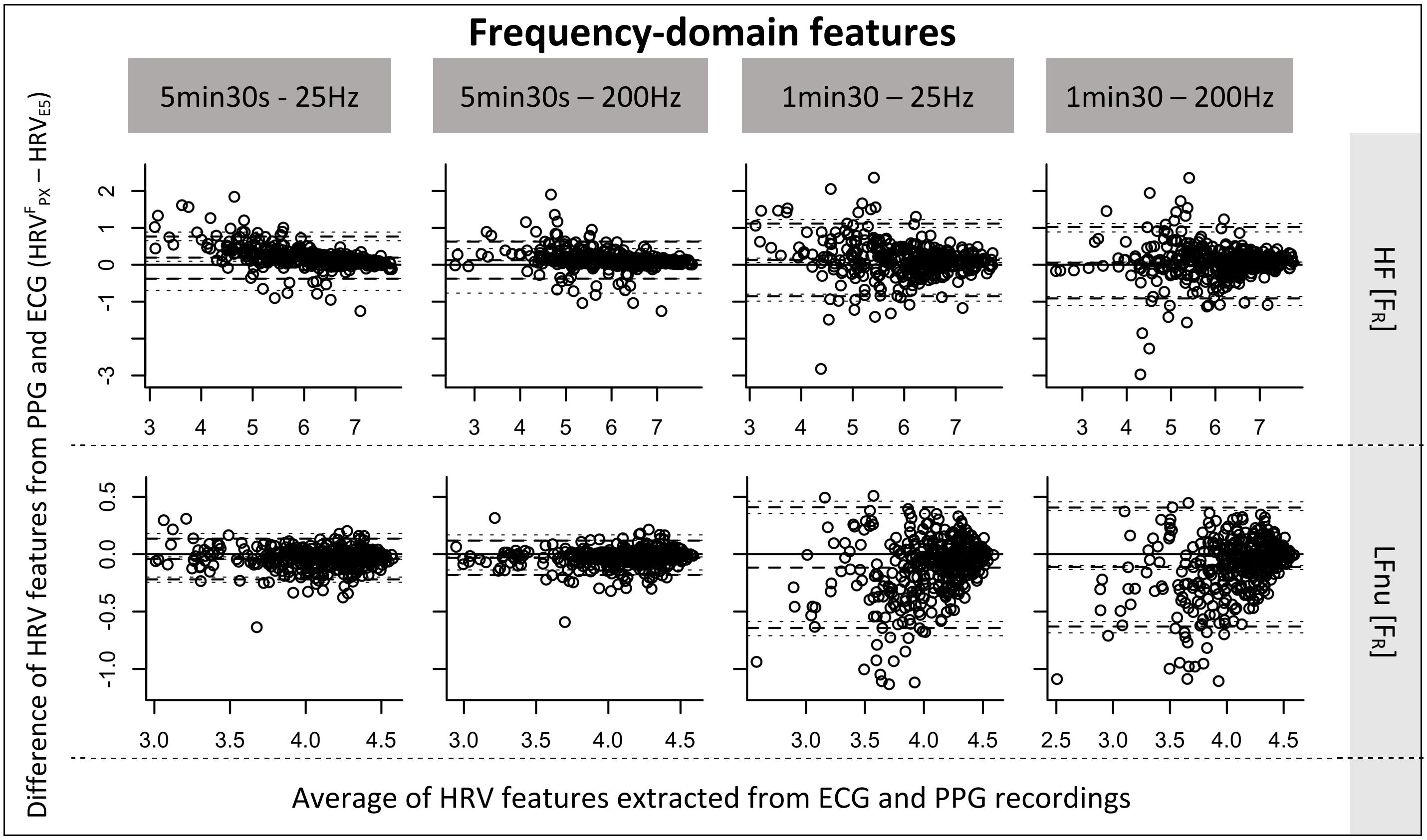

Frequency-domain features
5min30s - 25Hz
5min30s – 200Hz
1min30 – 25Hz
1min30 – 200Hz
HF [F$_R$]
LFnu [F$_R$]
Difference of HRV features from PPG and ECG (HRV$^F_{PX}$ – HRV$_{E5}$)
Average of HRV features extracted from ECG and PPG recordings

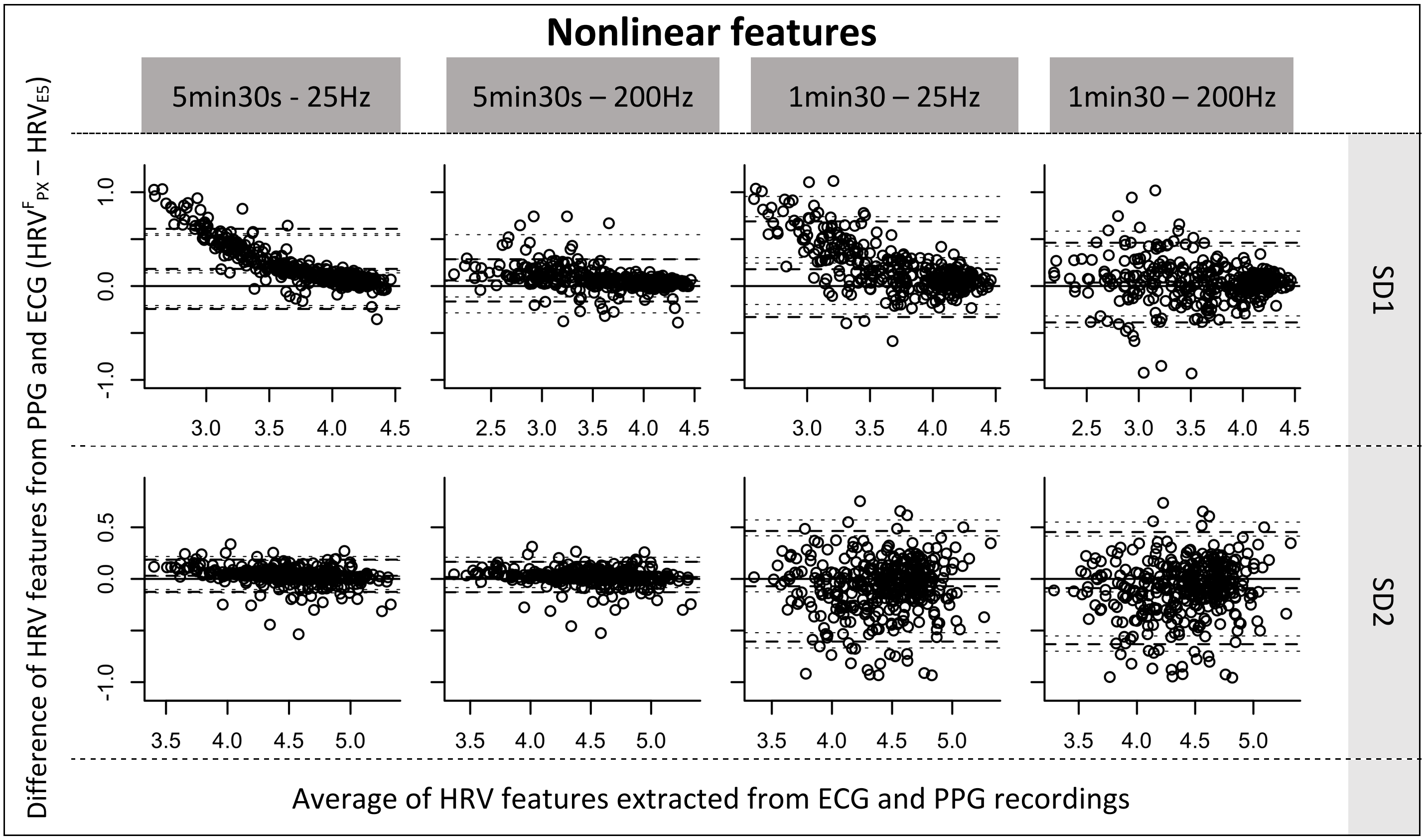

Nonlinear features
5min30s - 25Hz
5min30s – 200Hz
1min30 – 25Hz
1min30 – 200Hz
SD1
SD2
Difference of HRV features from PPG and ECG (HRV$^F_{PX}$ – HRV$_{E5}$)
Average of HRV features extracted from ECG and PPG recordings
